# Associations of Radiographic Cerebral Small Vessel Disease with Acute Intracerebral Hemorrhage Volume, Hematoma Expansion, and Intraventricular Hemorrhage

**DOI:** 10.1007/s12028-019-00876-4

**Published:** 2019-12-16

**Authors:** Simone M. Uniken Venema, Sandro Marini, H. Bart Brouwers, Andrea Morotti, Daniel Woo, Christopher D. Anderson, Jonathan Rosand

**Affiliations:** 1 Center for Genomic Medicine, Massachusetts General Hospital, Boston, MA USA; 2 Department of Neurology and Neurosurgery, Brain Center, University Medical Center Utrecht, Utrecht, The Netherlands; 3 Department of Neurology and Neurorehabilitation, IRCCS Mondino Foundation, Pavia, Italy; 4 Department Neurology and Rehabilitation Medicine, University of Cincinnati College of Medicine, Cincinnati, OH USA; 5 Division of Neurocritical Care and Emergency Neurology, Department of Neurology, Massachusetts General Hospital, Harvard Medical School, Boston, MA USA; 6 Henry and Allison McCance Center for Brain Health, Massachusetts General Hospital, Boston, MA USA

**Keywords:** Cerebral small vessel disease, Intracerebral hemorrhage, Leukoaraiosis, Cerebral atrophy, Computerized tomography

## Abstract

**Background and Objective:**

The aim of this study was to evaluate the impact of radiographic cerebral small vessel disease (CSVD) on the severity of acute intracerebral hemorrhage (ICH) as measured by: ICH volume, hematoma expansion, and extension of intraventricular hemorrhage (IVH).

**Methods:**

CSVD was determined on baseline computed tomography (CT) scans of patients from the Ethnic and Racial Variations of Intracerebral Hemorrhage study through the extent of leukoaraiosis and cerebral atrophy using visual rating scales. The associations of leukoaraiosis and atrophy with ICH volume, hematoma expansion, IVH presence, and severity of IVH were tested using multivariable regression models. Secondary analyses were stratified by hemorrhage location. Bonferroni correction was applied to correct for multiple testing.

**Results:**

A total of 2579 patients (mean age 61.7 years, 59% male) met inclusion criteria. Median ICH volume was 10.5 (Interquartile range [IQR] 4.0–25.3) mL. IVH was detected in 971 patients (38%). Neither leukoaraiosis nor atrophy was associated with hematoma expansion. Increasing grades of leukoaraiosis were associated with increased risk of IVH in a dose-dependent manner, while cerebral atrophy was inversely associated with IVH (both *P* for trend < 0.001). Increasing grades of global atrophy were dose-dependently associated with lower ICH volumes (ß (95% Confidence Interval [CI]) − 0.30[− 0.46, − 0.14], − 0.33[− 0.49, − 0.17], − 0.40[− 0.60, − 0.20], and − 0.54[− 0.76, − 0.32], for grades 1, 2, 3 and 4 compared to 0; all *P* < 0.001). The associations of leukoaraiosis with ICH volume were consistent with those of atrophy, albeit not meeting statistical significance.

**Conclusions:**

Leukoaraiosis and cerebral atrophy appear to have opposing associations with ICH severity. Cerebral atrophy correlates with smaller ICH volume and decreased risk and severity of IVH, while leukoaraiosis is associated with increased risk of IVH. Whether these observations reflect overlapping or divergent underlying mechanisms requires further study.

**Electronic supplementary material:**

The online version of this article (10.1007/s12028-019-00876-4) contains supplementary material, which is available to authorized users.

## Introduction

Spontaneous intracerebral hemorrhage (ICH) accounts for approximately 15% of acute strokes worldwide [1] and is associated with high rates of mortality and disability [2]. ICH is the most severe clinical manifestation of long-standing cerebral small vessel disease (CSVD), a disease of aging characterized by the accumulation of vascular risk factors [3]. The extent of CSVD at the time of ICH can be assessed using neuroimaging biomarkers, such as leukoaraiosis or cerebral atrophy measured on non-contrast computed tomography (CT) [4–6]. Individual variability in post-ICH outcome is influenced both by the extent of preexisting CSVD [7–9], as well as severity of the ICH itself. Hematoma volume is the most important prognostic determinant following ICH [10]. Intraventricular hemorrhage (IVH) occurring alongside the ICH doubles the risk of poor functional outcome at hospital discharge [11], and hematoma expansion, occurring in approximately one-third of patients, independently predicts poor outcome at 90 days post-stroke [12–14].

ICH is thought to arise from rupture of arteries with diminished microvascular integrity due to either hypertensive arteriopathy, affecting deep perforating arterioles, or cerebral amyloid angiopathy, affecting cortical and leptomeningeal vessels [3, 15]. Reflecting the distribution of the underlying type of microangiopathy, the former causes ICH’s in deep and infratentorial locations, while the latter is associated with ICH’s occurring in lobar regions [16, 17]. It has been hypothesized that the diffuse cerebral vasculopathies associated with CSVD might allow for more extensive acute-phase bleeding and therefore influence the cascade of events once an ICH has occurred, theoretically leading to larger hemorrhage volumes, risk of hemorrhage expansion, and more extensive IVH [18]. However, only one previous study has reported an association between leukoaraiosis and hematoma volume > 30 mL [19] and other studies found no association between leukoaraiosis and either hematoma volume [18, 20] or expansion [18, 20, 21]. The relationship between leukoaraiosis and IVH is also unclear [19, 22].

We hypothesize that the mechanisms underlying the association between preexisting pathologic burden of CSVD and poor outcome from ICH are likely to fall into two broad categories: (1) the effect of CSVD on the severity of the acute ICH, and (2) the effect of CSVD on the brain’s capacity for recovery. Our objective here is to address the first category, clarifying the relationships between two CT-based CSVD markers, leukoaraiosis and cerebral atrophy, and the severity of the acute ICH. Understanding the association of preexisting CSVD with the severity of ICH may help understand how CSVD impacts individual vulnerability in the setting of acute brain injury.

## Methods

### Study Population and Collection of Variables

Data were collected from the Ethnic and Racial Variations in Intracerebral Hemorrhage (ERICH) study, a multicenter prospective study of spontaneous ICH. As described elsewhere [23], the study enrolled a racially balanced, multi-ethnic cohort of 3000 primary ICH patients and matched controls. Trained study investigators obtained patient demographics and clinical data during hospital stay and thereafter, through case interviews with patients or their representatives, as well as through patient chart abstraction. Informed consent for participation in the ERICH study was obtained from patients or their legally authorized representatives. Institutional review boards from all participating sites approved the study protocols.

### Imaging Acquisition and Interpretation

For the current study, the first non-contrast CT performed upon admission was reviewed by trained investigators blinded to clinical data (SMUV, AM). Patients meeting the following imaging-related criteria were excluded from further analysis: (1) unreliable baseline CT scan due to poor imaging quality, (2) large hydrocephalus, severe midline shift or global edema which made the acquisition of imaging variables impossible, (3) multiple ICH, and (4) primary IVH. The remaining scans were used to obtain the following variables: ICH location, categorized as lobar versus non-lobar as previously done [24], ICH and IVH volumes, using semiautomated computerized volumetric analysis (Alice software, Parexel Corporation, Waltham, MA). The presence of IVH was defined as an IVH volume measurement of > 0. In patients with a follow-up CT scan, follow-up ICH volume was determined to assess hematoma expansion. Hematoma expansion was defined as a > 33% or > 6-mL increase in hematoma volume between baseline and first follow-up scan.

Using Analyze 11.0 software (AnalyzeDirect, Overland Park, KS) or Synedra software (Synedra Information Technologies, Innsbruck, Austria) the following variables were also determined: (1) extent of leukoaraiosis, (2) extent of cerebral atrophy and (3) the Graeb Score as a measure of IVH severity [25]. The extent of periventricular leukoaraiosis in anterior and posterior brain regions was graded according to a visual rating scale previously described by van Swieten and colleagues [26]. Anterior and posterior leukoaraiosis was graded as 0 (none), 1 (some) or 2 (severe), summing up to a total score ranging from 0 to 4 (Supplementary Fig. 1). The severity of atrophy was determined similarly using a visual rating scale [9], rated from 0 to 2 for central and cortical brain atrophy, again summing up to a total score ranging from 0 to 4 (Supplementary Fig. 1). Both leukoaraiosis and atrophy were assessed on the contralateral side of the ICH to avoid any influence of the hematoma itself on the ratings. The Graeb Score is based on presence of blood in the third, fourth, right lateral and left lateral ventricles and expansion of the individual ventricles, with a maximum score of 4 for each of the lateral ventricles and a maximum score of 2 for the third and fourth ventricles, when the ventricle is completely filled with blood and expanded. The total Graeb score is calculated by summing the individual scores, with a maximum total score of 12.

### Statistical Analysis

Categorical variables were described in percentages and continuous variables as mean and standard deviation (SD) or median and interquartile range, as appropriate. Demographic, admission, and imaging characteristics of patients with lobar and non-lobar ICH were compared using Chi-square tests for categorical variables and *t* tests or Mann–Whitney *U* tests for continuous variables. Variables that were associated with ICH volume, hematoma expansion, and IVH presence in univariate analyses were entered into multivariable models. Age, gender, and race ethnicity, as well as the CSVD variables, were forced into the models, whether or not they were significant in univariate analysis. Using stepwise backward regression, minimal multivariable models were built to investigate the associations between leukoaraiosis and atrophy with outcome variables of interest: (1) ICH volume, (2) hematoma expansion, (3) IVH presence, and (4) the Graeb Score, as a measure of IVH severity, in the subgroup of ICH patients with concomitant IVH. Binary logistic regression was used for models investigating hematoma expansion and IVH presence. Linear regression was used for models predicting ICH volume, which was natural log-transformed to approximate a normal distribution [24]. Ordinal logistic regression was used for models predicting the Graeb Score, which was categorized as some (Graeb Score of 1–3), modest (Graeb Score of 4–6), or severe (Graeb Score of > 7) and was treated as an ordered response variable. The proportional odds assumption was tested and was not violated. An alternative model, using log-transformed IVH volume, was used as an outcome variable in multiple linear regression to strengthen our findings related to IVH severity. In secondary analyses, the models for ICH volume and hematoma expansion were stratified by ICH location.

In all analyses, leukoaraiosis and atrophy were treated as ordered variables and each increase in atrophy and leukoaraiosis was compared to the reference category of 0. Multiple testing issues were addressed by applying a Bonferroni correction in all analyses, with a conservative *P* value of < 0.0063, based on eight independent tests, considered statistically significant. All analyses were performed using R statistical program [27].

## Results

### Inter-rater Agreement

The inter-rater agreement was determined by calculating an intraclass correlation coefficient (ICC), which was good for all measurements of CSVD markers (anterior leukoaraiosis: 0.78, 95% CI 0.63–0.87; posterior leukoaraiosis: 0.81, 95% CI 0.68–0.89; central atrophy: 0.87, 95% CI 0.79–0.93; cortical atrophy: 0.84, 95% CI 0.72–0.91). The ICC remained high throughout the study. The ICC for the Graeb score was also good (0.95, 95% CI 0.90–0.98).

### Study Participants and Baseline Characteristics

**Fig. 1:**
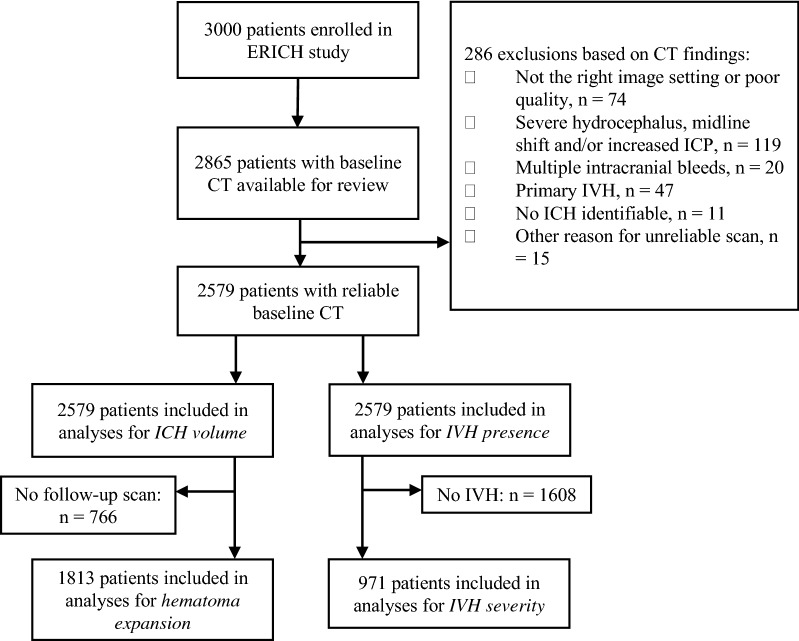
Flow chart of patients through the study selection process. *ERICH* ethnic and racial variations of intracerebral hemorrhage, *CT* computed tomography, *IVH* intraventricular hemorrhage, *ICH* intracerebral hemorrhage

A total of 2865 ICH patients included in the ERICH study had a baseline CT scan available for review. Of these, 286 patients met one or more of our exclusion criteria based on CT scan findings (Fig. 1). Of the 2579 patients included (mean age 61.7 [SD 14.5], 1541 male [59.8%]), 1805 (70.0%) had non-lobar ICH (Table 1). The median van Swieten score was 1 (IQR 0–2) and the median global atrophy score was 2 (IQR 0–2). ICH expansion occurred in 355 of 1813 (19.6%) patients for whom a baseline and follow-up CT scan was available. IVH was detected in 1018 patients (39.4%). Of these, 423 patients (41%) having mild IVH (Graeb Score 1–3), 371 patients (36%) having modest IVH (Graeb Score 4–6), and 229 (22%) having severe IVH (Graeb Score > 7). Frequency of expansion did not differ between lobar and non-lobar ICH.

**Table 1:** Baseline demographic and clinical characteristics

	All patients (*n* = 2579)
*Demographics*	
Age (mean [SD])	61.7 (14.5)
Male gender, *n* (%)	1541 (59.8)
Race/ethnicity (%)	
Black, *n*(%)	884 (34.3)
Hispanic, *n* (%)	876 (34.0)
White, *n* (%)	819 (31.8)
*Medical history*	
History of stroke, *n* (%)	551 (21.4)
Hypertension, *n* (%)	2074 (81.7)
Diabetes, *n* (%)	727 (28.7)
Alcohol use, *n* (%)	974 (37.8)
*Medication*	
Warfarin use (%)	219 (8.5)
Antiplatelet use (%)	701 (27.2)
*Admission variables*	
SBP (mean [SD])	184.8 (38.0)
BMI (median [IQR])	27.7 [24.1–32.6]
Admission GCS (median [IQR])	15.0 [11.0–15.0]
Admission glucose (mean [SD])	148.6 (64.6)
Time from ictus to baseline CT (%)	
< 6 h, *n* (%)	1355 (52.5)
> 6 h, *n* (%)	1094 (42.4)
Unknown, *n* (%)	130 (5.0)
*Hemorrhage characteristics*	
Non-lobar hemorrhage location, *n* (%)	1805 (70.0)
ICH volume in mL (median [IQR])	10.5 [4.0–25.3]
IVH presence, *n* (%)	1018 (39.4)
Graeb score, n (%)	
None (0)	1548 (60.0)
Some IVH (Graeb score 1–3)	423 (16.4)
Modest IVH (Graeb score 4–6)	371 (14.4)
Severe IVH (Graeb score > 7)	229 (8.9)
Undetermined	8 (0.3)
*CSVD variables*	
Leukoaraiosis, n (%)	
Grade 0	1065 (41.3)
Grade 1	447 (17.3)
Grade 2	447 (17.3)
Grade 3	265 (10.3)
Grade 4	329 (12.8)
Undetermined	26 (1.0)
Global atrophy, n (%)	
Grade 0	834 (32.3)
Grade 1	356 (13.8)
Grade 2	722 (41.8)
Grade 3	295 (11.4)
Grade 4	318 (12.3)
Undetermined	54 (2.1)

*BMI* body mass index; *CSVD* cerebral small vessel disease, *CT* computed tomography, *GCS* Glasgow Coma Score, *ICH* intracerebral hemorrhage, *IVH* intraventricular hemorrhage, *IQR* interquartile range, *SBP* systolic blood pressure, *SD* standard deviation

### ICH Volume and Hematoma Expansion

Age, male gender, Hispanic and white ethnicities, history of alcohol use, higher serum glucose, and presence of IVH were associated with larger ICH volume in multivariable models, whereas > 6 h from symptom onset to CT, a non-lobar ICH location, increasing body mass index, and history of stroke were associated with smaller ICH volume (Supplementary Table 1).

**Fig. 2:**
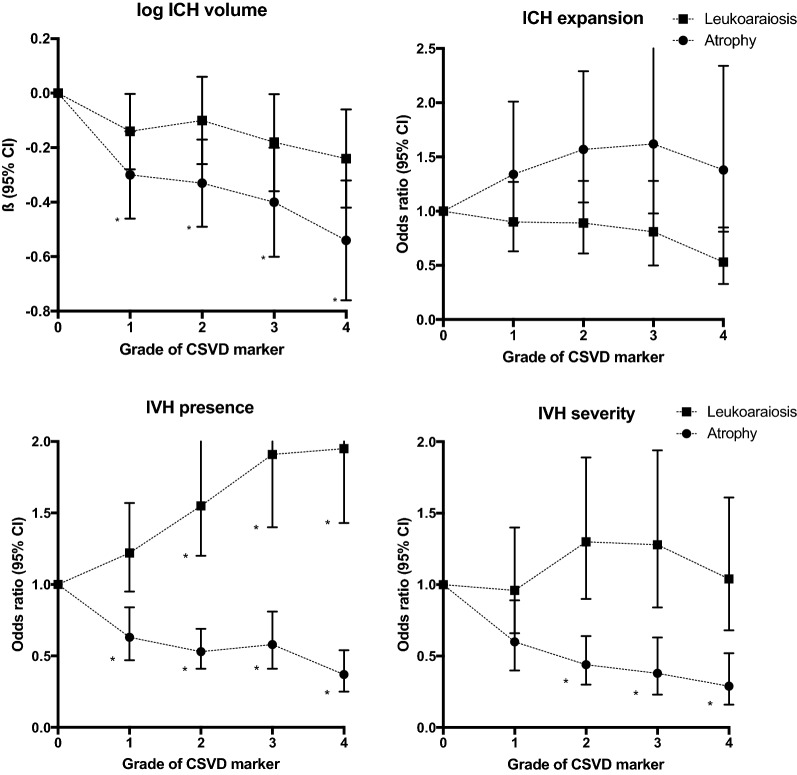
Associations of increasing extent of CSVD variables and log-transformed ICH volume (upper left), ICH expansion (upper right), IVH presence (lower left) and the Graeb Score (lower right). All models were adjusted for age, gender, and race ethnicity. In addition, analysis for ICH volume was adjusted for history of stroke, history of alcohol use, time from symptoms to CT, ICH location, and presence of IVH. Analysis for ICH expansion was adjusted for history of stroke, warfarin use, platelet count on admission, time from symptoms to CT, and ICH volume. Analysis for IVH presence was adjusted for serum glucose, ICH location, ICH volume. Analysis for the Graeb Score was adjusted for admission serum glucose. *P* values considered significant after applying Bonferroni correction are indicated with an asterisk (*). *ICH* intracerebral hemorrhage, *CSVD* cerebral small vessel disease, *IVH* intraventricular hemorrhage

In univariate modeling, all grades of cerebral atrophy were associated with smaller ICH volume, with the strongest association seen for severe (grade 4) atrophy (Supplementary Table 1). After adjusting for potential confounders, all grades of cerebral atrophy were still, in a dose-dependent manner, associated with smaller ICH volume (Table 2, Fig. 2, Supplementary Table 1). Similarly, all grades of leukoaraiosis were associated with smaller ICH volume in a dose-dependent manner in univariate analysis (Supplementary Table 1). In the multivariate model, the dose dependence of these associations was largely retained, albeit not meeting statistical significance after applying Bonferroni correction (Table 2, Fig. 2, Supplementary Table 1). In multivariate analyses stratified by ICH location, cerebral atrophy was, in a dose-dependent manner, associated with lower ICH volume in non-lobar ICH only (*P* for trend < 0.001). In lobar ICH, only grade 3 atrophy was significantly associated with lower ICH volume (Supplementary Table 3). Leukoaraiosis was not significantly associated with ICH volume in either lobar or non-lobar ICH after stratification (Supplementary Table 3).

**Table 2:** Associations of increasing extent of CSVD variables and log-transformed ICH volume, ICH expansion, IVH presence, and the Graeb Score

CSVD variable	Log ICH volume (*n* = 2579)		ICH expansion (*n* = 1813)		IVH presence (*n* = 2579)		Graeb Score (*n* = 971)	
	Adjusted ß (95% CI)	*P* value	Adjusted OR (95% CI)	*P* value	Adjusted OR (95% CI)	*P* value	Adjusted OR (95% CI)	*P* value
*Leukoaraiosis*								
Grade 0	Reference	–	Reference	–	Reference	–	Reference	–
Grade 1	− 0.14 (− 0.28, − 0.003)	0.061	0.90 (0.63–1.27)	0.539	1.22 (0.95–1.57)	0.123	0.96 (0.66–1.40)	0.844
Grade 2	− 0.10 (–0.26, 0.06)	0.187	0.89 (0.61–1.28)	0.540	1.55 (1.20–2.01)	0.001*	1.30 (0.90–1.89)	0.160
Grade 3	− 0.18 (− 0.36, − 0.004)	0.056	0.81 (0.50–1.28)	0.379	1.91 (1.40–2.61)	<0.001*	1.28 (0.84–1.94)	0.249
Grade 4	− 0.24 (− 0.42, − 0.06)	0.009	0.53 (0.33–0.85)	0.010	1.95 (1.43–2.65)	<0.001*	1.04 (0.68–1.61)	0.850
*Global atrophy*								
Grade 0	Reference	–	Reference	–	Reference	–	Reference	–
Grade 1	− 0.30 (− 0.46, − 0.14)	<0.001*	1.34 (0.89–2.01)	0.156	0.63 (0.47–0.84)	0.002*	0.60 (0.40–0.89)	0.012
Grade 2	− 0.33 (− 0.49, − 0.17)	<0.001*	1.57 (1.08–2.29)	0.018	0.53 (0.41–0.69)	<0.001*	0.44 (0.30–0.64)	<0.001*
Grade 3	− 0.40 (− 0.60, − 0.20)	<0.001*	1.62 (0.98–2.65)	0.058	0.58 (0.41–0.81)	0.002*	0.38 (0.23–0.63)	<0.001*
Grade 4	− 0.54 (− 0.76, − 0.32)	<0.001*	1.38 (0.81–2.34)	0.236	0.37 (0.25–0.54)	<0.001*	0.29 (0.16–0.52)	<0.001*

All models were adjusted for age, gender, and race ethnicity. In addition, analysis for ICH volume was adjusted for history of stroke, history of alcohol use, time from symptoms to CT, ICH location, presence of IVH. Analysis for ICH expansion was adjusted for history of stroke, warfarin use, platelet count on admission, time from symptoms to CT, and ICH volume. Analysis for IVH presence was adjusted for serum glucose, ICH location, ICH volume. Analysis for the Graeb Score was adjusted for admission serum glucose

*CI* class interval; *CSVD* cerebral small vessel disease, *ICH* intracerebral hemorrhage, *IVH* intraventricular hemorrhage, *OR* odds ratio

*P values considered significant after applying Bonferroni correction. Full models are presented in Supplementary Tables

Neither leukoaraiosis nor cerebral atrophy was independently associated with hematoma expansion in both univariate and multivariable analyses after applying Bonferroni correction (Table 2, Fig. 2, Supplementary Table 2). Variables independently associated with ICH expansion in multivariable models included warfarin use, history of stroke, and larger baseline ICH volume, whereas platelet count on admission, time from symptoms to CT, and IVH presence were associated with decreased risk of hematoma expansion. Results remained unchanged after stratifying the analyses by ICH location (Supplementary Table 4).

### IVH Presence and Severity

Age, Hispanic ethnicity, increasing serum glucose level, larger ICH volume, and non-lobar ICH location were independently associated with IVH presence in multivariable logistic regression (Supplementary Table 5). In patients with ICH and concomitant IVH, increasing serum glucose level was associated with a higher Graeb Score in multivariable ordinal logistic regression models (Supplementary Table 6).

The extent of cerebral atrophy was associated with lower risk of IVH presence in both univariate and multivariate analyses, with the strongest association seen for severe (grade 4) atrophy (Fig. 2, Table 2, Supplementary Table 5). Similarly, increasing extent of cerebral atrophy was associated with lower IVH severity in a dose-dependent manner, in both univariate and multivariate analysis (both *P* for trend < 0.001) (Fig. 2, Table 2, Supplementary Table 6).

In contrast, increasing grades of leukoaraiosis were associated with IVH presence in a dose-dependent manner (*P* for trend < 0.001) in both univariate and multivariate analysis (Table 2, Fig. 2, Supplementary Table 5). The extent of leukoaraiosis was not associated with the Graeb Score in either univariate or multivariate modeling (Fig. 2, Table 2, Supplementary Table 6). Findings for the alternative analyses for IVH severity, using log-transformed IVH volume as an outcome variable in multivariable linear models, were similar regarding the association of CSVD variables with IVH volume (data not shown).

## Discussion

Our study aims to elucidate how long-standing CSVD affects individual vulnerability in the setting of acute ICH by investigating the associations of leukoaraiosis and cerebral atrophy with ICH volume, hematoma expansion, and presence and severity of concomitant IVH. The study demonstrates that more extensive cerebral atrophy was associated with lower ICH volume. The associations of leukoaraiosis with ICH volume were consistent with those of atrophy, albeit not meeting statistical significance. In contrast, the associations of leukoaraiosis and atrophy with IVH occurrence went in opposite directions, with increase in extent of cerebral atrophy being associated with less frequent occurrence of IVH and less severe IVH, while leukoaraiosis was associated an increased risk of IVH occurrence.

In line with previous studies that used magnetic resonance imaging (MRI) to assess white matter lesions, we did not find an association between leukoaraiosis and either ICH volume or hemorrhage expansion [18, 20, 21]. We extend these previous results by showing that leukoaraiosis was, in a dose-dependent manner, associated with an increased risk of IVH, but not with IVH severity. Only two prior studies have investigated this relationship, one of which reported a positive association between leukoaraiosis and increased odds of IVH presence [22]. The other study found no such association [19]. The current study benefits from a larger patient population, the fact that two distinct measurements of IVH severity were used to increase the reliability of our results, and the fact that we studied two different biomarkers of CSVD.

Vasculopathies in the context of subcortical CSVD are characterized by arteriolar lipohyalinosis and venous collagenosis, both causing concentric thickening of the vessel wall [15, 28], and fewer vessels per unit of tissue [29]. This could explain why patients with advanced CSVD were less prone to severe acute-phase bleeding, contrary to previous hypotheses [18]. Alternatively, patients with extensive CSVD might be more easily symptomatic from small bleeds, while patients with a low burden of CSVD may be more likely to have a clinically silent ICH that remains undetected. Third, patients with smaller cerebral volumes may suffer from smaller hematomas accordingly, since there is simply less parenchyma for the hematoma to occupy. The opposite effect of leukoaraiosis and cerebral atrophy on the risk of IVH occurrence suggests that despite being considered two distinct phenotypes reflecting similar underlying pathology [30], their overall clinical effect may vary depending on poorly characterized factors, such as coincident pathologies impacting these radiographic markers.

The current study increases our understanding of the relationship between CSVD and outcome after ICH. Although we did not investigate outcome following ICH, the burden of CSVD has consistently been shown to be associated with poor outcome in both ischemic and hemorrhagic stroke [7–9]. Our results suggest this well-established relationship is not mediated by the acute complications of the ICH itself, such as intraventricular extension of the hematoma, initial hematoma volume, or hemorrhage expansion. Hence, chronic CSVD and features of the acute ICH itself likely cause poor outcome through distinct mechanisms. This is in line with one previous study, which attempted to disentangle the complicated relationships between preexisting CSVD, features of the ICH itself and poor outcome following ICH, reaching a similar conclusion that the acute manifestation of an ICH does not mediate the relationship between CSVD and poor outcome [20].

The current study has several strengths and limitations. Strengths include our multi-ethnic study population and large sample size, central adjudication of outcome assessment and our high inter-rater agreement for the neuroimaging markers. Limitations include the fact that, despite a homogeneous radiographic appearance of CSVD markers on neuroimaging, our chosen biomarkers have been shown to correlate with heterogeneity of CSVD lesions on histopathological examination, and thus, it is unclear how well the imaging markers reflect the true pathological disease burden of CSVD [15, 31]. This could be further impacted by our use of CT-based CSVD biomarkers, which demonstrate white matter lesions only in more advanced stages of CSVD and we are unable to determine the importance of early-stage CSVD which does not yet appear on CT as leukoaraiosis or atrophy [32]. Nevertheless, the dose dependency of the associations found in this study suggests that our findings are not based on chance alone and that visual rating of CT-based CSVD phenotypes likely reflects underlying pathology. CT is more widely available and is used routinely in the setting of acute stroke, boosting our study’s power relative to MRI-based phenotypes. Moreover, studies show substantial agreement between MRI and CT-based visual rating scales for leukoaraiosis and atrophy [5, 33]. A second limitation is related to the possibility of limited external validity: patients with lethally large hematoma volumes at baseline, or a severe clinical manifestation (low GCS), may have been less likely to be included in the study due to the necessity for immediate intervention, early deaths, and the fact that patients with large hydrocephalus or midline shift on baseline CT were excluded from this study. Therefore, the results of the current study might not be directly generalizable to patients with a severe manifestation of acute ICH. Moreover, as the CSVD scores are unevenly distributed in our sample, this could have negatively impacted resolution of effects for some of the ordinal values, although this is mitigated by our demonstration of dose dependence.

## Conclusions

We demonstrate that CT-based markers of CSVD differentially associated with the initial severity and acute complications of an ICH. Whereas cerebral atrophy was associated with smaller ICH volume, lower risk of IVH presence, and less severe IVH, the associations of leukoaraiosis were limited to an increased risk of IVH presence. We conclude that the association of preexisting CSVD with poor outcome after ICH is likely to be mediated by mechanisms that are independent of the manifestation of the acute ICH itself. Future studies are needed to confirm our findings and elucidate the underlying biological mechanisms that mediate the relationship between CSVD and poor outcome following ICH.

## Electronic supplementary material

Below is the link to the electronic supplementary material.

Supplementary material 1 (DOCX 565 kb)
