# Supplementary material for: Associations of Radiographic Cerebral Small Vessel Disease with Acute Intracerebral Hemorrhage Volume, Hematoma Expansion, and Intraventricular Hemorrhage
Source: Neurocrit Care. 2019 Dec 16;32(2):383–91. doi: 10.1007/s12028-019-00876-4 (PMC7082383; doi:10.1007/s12028-019-00876-4)

**SUPPLEMENTARY MATERIALS**

| **Supplementary table 1: univariate and multivariate linear regression for log-transformed ICH volume (n = 2579).** | | | | |
| --- | --- | --- | --- | --- |
| CSVD marker | Unadjusted ß  (95% CI) | P value | Adjusted ß  (95% CI) | P value |
| Leukoaraiosis  Grade 0  Grade 1  Grade 2  Grade 3  Grade 4 | Reference  -0.14 (-0.31, 0.03)  -0.19 (-0.33, -0.06)  -0.23 (-0.41, -0.05)  -0.31 (-0.49, -0.13) | 0.062  0.012  0.014  <0.001* | Reference  -0.14 (-0.28, -0.003)  -0.10 (-0.26, 0.06)  -0.18 (-0.36, -0.004)  -0.24 (-0.28, -0.06) | 0.061  0.187  0.056  0.009 |
| Global atrophy  Grade 0  Grade 1  Grade 2  Grade 3  Grade 4 | Reference  -0.34 (-0.49, -0.19)  -0.28 (-0.43, -0.13)  -0.21 (-0.39, -0.03)  -0.47 (-0.64, -0.30) | <0.001*  <0.001*  0.022  <0.001* | Reference  -0.30 (-0.46, -0.14)  -0.33 (-0.49, -0.17)  -0.40 (-0.60, -0.20)  -0.54 (-0.76, -0.32) | <0.001*  <0.001*  <0.001*  <0.001* |

ICH, intracerebral hemorrhage; CT, computed tomography; IVH, intraventricular hemorrhage; CI, confidence interval. Adjusted for age, gender, race-ethnicity, history of stroke, BMI, history of alcohol use, time from symptoms to CT, ICH location, presence of IVH, and CSVD markers.

*P-values considered significant after applying Bonferroni correction.

| **Supplementary table 2: univariate and multivariate binary logistic regression for hematoma expansion (n = 1813).** | | | | |
| --- | --- | --- | --- | --- |
|  | Unadjusted OR  (95% CI) | P value | Adjusted OR  (95% CI) | P value |
| Leukoaraiosis  Grade 0  Grade 1  Grade 2  Grade 3  Grade 4 | Reference  0.97 (0.70-1.33)  0.97 (0.70-1.34)  0.85 (0.55-1.28)  0.62 (0.41-0.92) | -  0.849  0.872  0.448  0.022 | Reference  0.90 (0.63-1.27)  0.89 (0.61-1.28)  0.81 (0.50-1.28)  0.53 (0.33-0.85) | -  0.539  0.540  0.379  0.010 |
| Global atrophy  Grade 0  Grade 1  Grade 2  Grade 3  Grade 4 | Reference  1.40 (0.96-2.02)  1.39 (1.03-1.89)  1.50 (1.01-2.21)  1.16 (0.77-1.72) | -  0.076  0.033  0.042  0.470 | Reference  1.34 (0.89-2.01) 1.57 (1.08-2.29)  1.62 (0.98-2.65)  1.38 (0.81-2.34) | -  0.156  0.018  0.058  0.236 |

CT, computed tomography; IVH, intraventricular hemorrhage; ICH, intracerebral hemorrhage. Adjusted for age, gender, race-ethnicity, history of stroke, warfarin use, admission platelet count, time from symptoms to CT, baseline ICH volume, presence of IVH, and CSVD markers. *P-values considered significant after applying Bonferroni correction.

| **Supplementary table 3: multivariable linear regression for log-transformed ICH volume, stratified by ICH location.** | | | | |
| --- | --- | --- | --- | --- |
|  | **Non-lobar ICH (n = 1805)** | | **Lobar ICH (n = 774)** | |
|  | Adjusted ß (95% CI) | P value | Adjusted ß (95% CI) | P value |
| Leukoaraiosis  Grade 0  Grade 1  Grade 2  Grade 3  Grade 4 | Reference  -0.14 (-0.32, 0.04)  -0.03 (-0.21, 0.15)  -0.08 (-0.30, 0.14)  -0.19 (-0.41, 0.03) | -  0.116  0.787  0.488  0.096 | Reference  -0.14 (-0.39, 0.11)  -0.29 (-0.56, -0.02)  -0.41 (-0.74, -0.08)  -0.32 (-0.61, -0.03) | -  0.300  0.030  0.013  0.036 |
| Global atrophy  Grade 0  Grade 1  Grade 2  Grade 3  Grade 4 | Reference  -0.26 (-0.46, -0.06)  -0.32 (-0.50, -0.14)  -0.33 (-0.58, -0.08)  -0.53 (-0.80, -0.26) | -  0.010  <0.001*  0.010  <0.001* | Reference  -0.42 (-0.71, -0.13)  -0.32 (-0.59, -0.05)  -0.54 (-0.87, -0.21)  -0.50 (-0.85, -0.15) | -  0.005*  0.018  0.001*  0.006* |

ICH, intracerebral hemorrhage; CT, computed tomography; IVH, intraventricular hemorrhage. Adjusted for age, gender, race-ethnicity, history of stroke, history of alcohol use, BMI, time from symptoms to CT, ICH location, presence of IVH, and CSVD markers. *P-values considered significant after applying Bonferroni correction.

| **Supplementary table 4: multivariable logistic regression for hematoma expansion, stratified by ICH location.** | | | | |
| --- | --- | --- | --- | --- |
|  | **Non-lobar ICH (n = 1321)** | | **Lobar ICH (n = 492)** | |
|  | Adjusted OR (95% CI) | P value | Adjusted OR (95% CI) | P value |
| Leukoaraiosis  Grade 0  Grade 1  Grade 2  Grade 3  Grade 4 | Reference  0.91 (0.60-1.36)  0.97 (0.63-1.48)  0.88 (0.50-1.51)  0.59 (0.32-1.02) | -  0.635  0.904  0.653  0.067 | Reference  0.89 (0.43-1.79)  0.60 (0.27-1.27)  0.64 (0.24-1.56)  0.45 (0.18-1.07) | -  0.752  0.192  0.344  0.082 |
| Global atrophy  Grade 0  Grade 1  Grade 2  Grade 3  Grade 4 | Reference  1.45 (0.91-2.3)  1.51 (0.98-2.33)  1.41 (0.75-2.58)  1.40 (0.74-2.64) | -  0.117  0.063  0.276  0.299 | Reference  0.95 (0.38-2.27)  1.67 (0.78-3.64)  2.10 (0.83-5.31)  1.18 (0.42-3.25) | -  0.909  0.189  0.117  0.745 |

CT, computed tomography; IVH, intraventricular hemorrhage; ICH, intracerebral hemorrhage. Adjusted for age, gender, race-ethnicity, history of stroke, warfarin use, admission platelet count, time from symptoms to CT, baseline ICH volume, presence of IVH, and CSVD markers. *P-values considered significant after applying Bonferroni correction.

| **Supplementary table 5: univariate and multivariate binary logistic regression for IVH presence (n = 2579).** | | | | |
| --- | --- | --- | --- | --- |
|  | Unadjusted OR (95% CI) | P value | Adjusted OR (95% CI) | P value |
| Leukoaraiosis  Grade 0  Grade 1  Grade 2  Grade 3  Grade 4 | Reference  1.11 (0.88-1.39)  1.30 (1.04-1.63)  1.57 (1.19-2.06)  1.51 (1.17-1.94) | -  0.369  0.022  0.001*  0.001* | Reference  1.22 (0.95-1.57)  1.55 (1.20-2.01)  1.91 (1.40-2.61) 1.95 (1.43-2.65) | -  0.123 0.001* <0.001* <0.001* |
| Global atrophy  Grade 0  Grade 1  Grade 2  Grade 3  Grade 4 | Reference  0.66 (0.51-0.86)  0.68 (0.56-0.84)  0.77 (0.58-1.00)  0.60 (0.46-0.79) | -  0.003*  <0.001*  0.0540  <0.001* | Reference 0.63 (0.47-0.84) 0.53 (0.41-0.69) 0.58 (0.41-0.81) 0.37 (0.25-0.54) | - 0.002* <0.001* 0.002* <0.001* |

CT, computed tomography; IVH, intraventricular hemorrhage; ICH, intracerebral hemorrhage. Adjusted for age, gender, race-ethnicity, serum glucose, ICH location, ICH volume and CSVD markers. *P-values considered significant after applying Bonferroni correction.

| **Supplementary table 6: univariate and multivariate ordinal logistic regression for the Graeb Score (n = 971).** | | | | |
| --- | --- | --- | --- | --- |
|  | Unadjusted OR (95% CI) | P value | Adjusted OR (95% CI) | P value |
| Leukoaraiosis  Grade 0  Grade 1  Grade 2  Grade 3  Grade 4 | Reference  0.81 (0.57-1.14)  0.99 (0.71-1.38)  0.92 (0.63-1.34)  0.77 (0.53-1.11) | -  0.225  0.935  0.661  0.160 | Reference  0.96 (0.66-1.40)  1.30 (0.90-1.89)  1.28 (0.84-1.94)  1.04 (0.68-1.61) | -  0.844  0.160  0.249  0.850 |
| Global atrophy  Grade 0  Grade 1  Grade 2  Grade 3  Grade 4 | Reference  0.62 (0.42-0.91)  0.50 (0.37-0.68)  0.42 (0.27-0.63)  0.34 (0.21-0.53) | -  0.014  <0.001*  <0.001*  <0.001* | Reference  0.60 (0.40-0.89)  0.44 (0.30-0.64)  0.38 (0.23-0.63) 0.29 (0.16-0.52) | -  0.012  <0.001*  <0.001* <0.001* |

CT, computed tomography; IVH, intraventricular hemorrhage; ICH, intracerebral hemorrhage. Graeb Score categorized as some (1-3), modest (4-6) and severe (>7). Model was adjusted for age, gender, race-ethnicity, admission serum glucose, and CSVD markers.

*P-values considered significant after applying Bonferroni correction.

**Supplementary figure 1: Visual rating scale for determining extent of global atrophy and leukoaraiosis on CT imaging.** Central (upper row) and cortical (lower row) atrophy were graded 0 (none, left), 1 (modest amount, middle) or 2 (severe, right), summing up to a total maximum score of 4. Anterior and posterior periventricular leukoaraiosis were similarly graded.


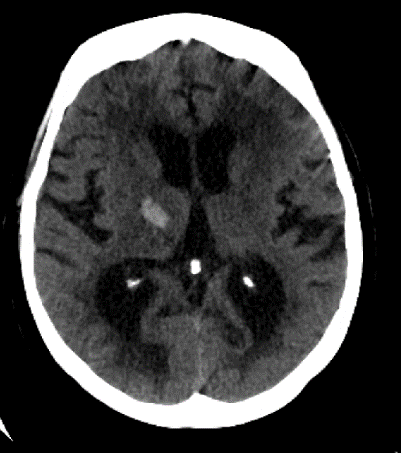

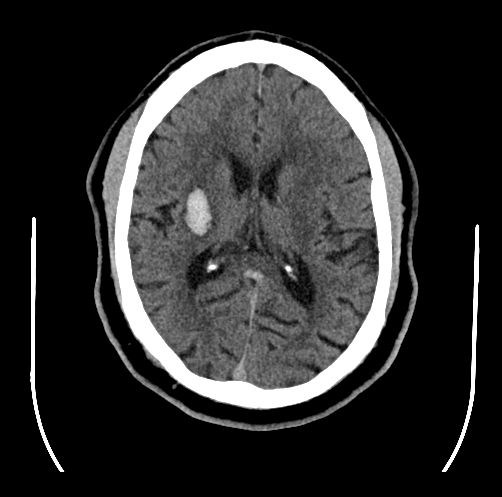

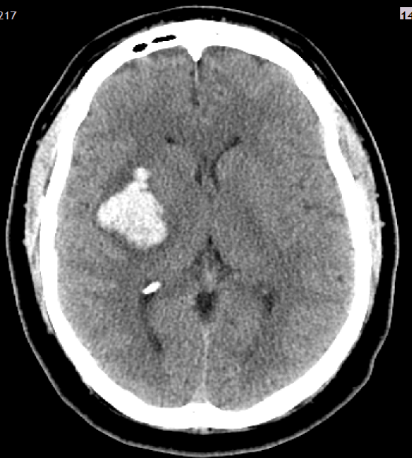


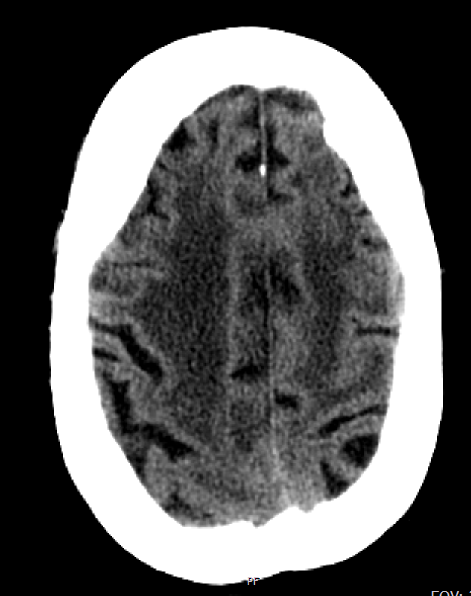

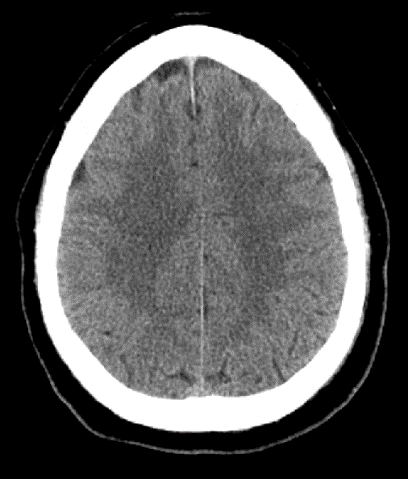

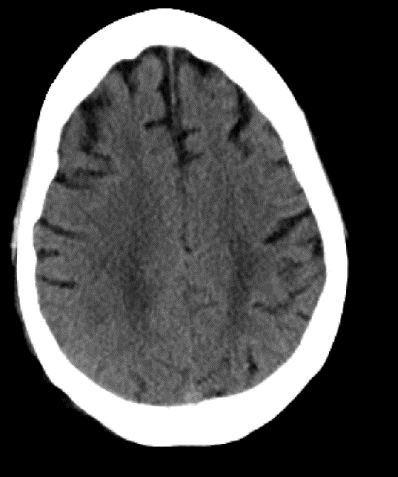

Supplement: Supplementary file 1 — Supplementary material 1 (DOCX 565 kb) [file 12028_2019_876_MOESM1_ESM.docx]
